# Evaluation of preoperative calculation methods of osteotomy size in ankylosing spondylitis with thoracolumbar or lumbar kyphosis

**DOI:** 10.1186/s12891-022-06043-9

**Published:** 2022-12-09

**Authors:** Jie Cheng, Shuwen Zhang, Weibin Sheng

**Affiliations:** 1 Department of Orthopedic Surgery, Affiliated Hospital of Zunyi Medical University, Zunyi, 563000 Guizhou China; 2 Department of Orthopedic, People’s Hospital of Xinjiang Uygur Autonomous Region, Urumchi, 830001 Xinjiang China; 3 Department of Spine Surgery, the First Affiliated Hospital of Xinjiang Medical University, 137 Liyushan Avenue, Xinshi District, Urumqi, 830054 Xinjiang China

**Keywords:** Ankylosing spondylitis, Thoracolumbar kyphosis, Osteotomy angle, Preoperative calculation method

## Abstract

**Background:**

To evaluate the accuracy of different preoperative calculation methods of osteotomy size in ankylosing spondylitis with thoracolumbar or lumbar kyphosis and analyze its clinical significance.

**Methods:**

Twenty-two cases of AS patients with thoracolumbar or lumbar kyphosis, from January 2015 to December 2018, who underwent one-level SPO surgery in our hospital, were retrospectively reviewed. The sagittal parameters were measured at pre-operation and last follow up using Surgimap software, and theoretical values of sagittal parameters were calculated according to pre-operative PI. The osteotomy angles of different methods were measured using Surgimap software. Paired *t* test was used to for the statistical analysis.

**Results:**

The mean follow-up time of all patients was 30.00 $\pm$ 3.56 months. The osteotomy sites were located at T12 in 3 cases, L1 in 6 cases, L2 in 9 cases, and L3 in 4 cases. Compared to pre-operative sagittal parameters, post-operative PT, SS, LL, and SVA were significantly improved (*P* $<$ 0.05). Compared to the OVA (46.57 $\pm$ 2.32 $^\circ$), there was a significantly larger angle predicted by Surgimap method (53.80 $\pm$ 9.79 $^\circ$), CAM-HA method (56.61 $\pm$ 8.58 $^\circ$), and HP-HA method (60.07 $\pm$ 13.58 $^\circ$), respectively (*P* $<$ 0.05). But no significant difference was found between the postoperative osteotomy angle and those of SFA method (51.24 $\pm$ 12.14 $^\circ$) and FBI method (48.08 $\pm$ 12.49 $^\circ$) (*P* $>$ 0.05).

**Conclusion:**

For AS patients with thoracolumbar or lumbar kyphosis, the SFA method, FBI method, and Surgimap method can be used to predict the osteotomy angle precisely, however, considering the rationality of parameter settings and the operability, SFA method is relatively more suitable for such population.

## Introduction

Ankylosing spondylitis (AS) is a chronic immune-mediated inflammatory arthritis, mainly affecting sacroiliac joint and the axial skeleton. The prevalence of AS ranged from 0.1% to 0.4%, usually in males around the age of 30 [1]. Previous studies reported that more than 30% of AS patients were accompanied with thoracolumbar or lumbar kyphosis [2]. The sagittal imbalance leads to difficulty in lying down flat, standing, sitting and walking, loss of horizontal visual axis, abdominal compression and impaired respiratory function in severe cases, which negatively affect health-related quality of life (HRQOL) in patients with AS [3]. Therefore, surgical intervention is often necessary for these patients to restore the sagittal alignment of the spine.

Spinal osteotomy is a widely used surgical technique to correct kyphosis deformity and to improve the spino-pelvic sagittal alignment. Among them, the Smith-Peterson osteotomy (SPO) and pedicle subtraction osteotomy (PSO) are the two most common surgical methods, by which can obtain satisfactory correction angle [4]. However, the unproper preoperative osteotomy scheme often results in inadequate or excessive correction after surgery, with poor clinical outcomes [5]. In 2006, Ondra et al. [6] proposed a trigonometric method to determine the degree of PSO needed for correction of sagittal deformity. Soon afterwards, Yang et al. [7] found that the trigonometric method was an approximation and proposed a modified method for calculating the exact angle required for PSO. However, the biggest drawback of both methods was that they regarded the spine as a rigid body and neglected the compensatory effects of the body. With the further understanding of the global spine-pelvis-lower limb sagittal balance and its compensatory mechanisms, scholars have consecutively proposed different prediction plans of preoperative osteotomy angle, such as, center of both acoustic meati-hip axis (CAM-HA) method [8], full balance integrated technique (FBI) [9], the spino-femoral angle method (SFA) [10], Surgimap method [11], hilus pulmonis-hip axis (HP-HA) method [12], and so on.

By reviewing the literatures published, obvious differences were found regarding the sagittal parameters, compensatory mechanisms, and evaluation of compensatory mechanisms abovementioned methods. In addition, some methods even lacked clinical verifications. So far, reports on the accuracy and availability of different methods are scarce. Therefore, this study aimed to evaluate the accuracy of different osteotomy angle prediction methods and explore the clinical significance of different methods.

## Materials and methods

### Patient population

This retrospective study enrolled patients with AS treated at our hospital between January 2015 and December 2018. Patient records were provided by the hospital database.

Inclusion criteria were as follows: (1) AS patients had thoracolumbar or lumbar kyphosis deformity, with or without coronal imbalance; (2) Patients underwent single-level PSO treatment; (3) ODI scores were less than 20 and SVA was less than 7 cm at the last follow-up; (4) The follow-up time was more than 2 years. Exclusion criteria were: (1) Patients with flexion contracture deformity of the hip; (2) AS patients undergoing two-level PSO surgery; (3) Prior spinal surgery; (4) AS patients complicated by spinal fracture, tuberculosis, or tumor diseases. According to inclusion and exclusion criteria, 22 cases were eventually enrolled in this study.

### Sagittal parameters

**Fig. 1:**
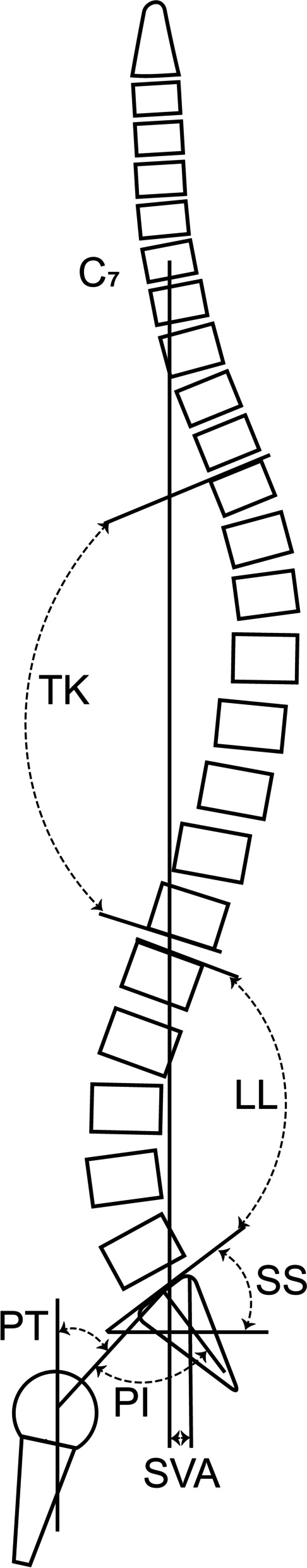
Measurement of spino-pelvis parameters. Sagittal vertical axis (SVA) was defined as the horizontal distance from the posterosuperior corner of S1 to the C7 plumb line. Thoracic kyphosis (TK) was measured from the superior endplate of T5 to the inferior endplate of T12. Lumbar lordosis (LL) was measured from the superior endplate of L1 to the superior endplate of S1. Pelvis incidence (PI) was defined as the angle subtended by a line drawn between the center of the femoral head and the sacral endplate and a line drawn perpendicular to the center of the sacral endplate. Pelvis tilt (PT) was defined as the angle subtended by a line drawn from the midpoint of the sacral endplate to the center of the bi-coxo-femoral axis and a vertical plumb line extended from the bi-coxo-femoral axis. Sacral slope (SS) was defined as the angle subtended by a line drawn along the endplate of the sacrum and a horizontal reference line extended from the posterior superior corner of S1

Radiographic data collection consisted of full-length, standing sagittal radiographs obtained in free-standing posture with hand placed on supports and shoulders in 45 $^\circ$ of forward elevation. The following spinopelvic parameters were measured using Surgimap software (Fig. 1).

The sagittal vertical axis (SVA) was defined as the horizontal offset from the posterosuperior corner of S1 to the C7 plumb line. The distance was noted positive when the C7 plumb line projection was anterior to the posterior corner of the sacral endplate and negative when the projection of that line lied behind the posterior corner of the sacrum. The thoracic kyphosis (TK) was measured from the superior endplate of T5 vertebrae to the inferior plate of T12 (Positive when in kyphosis). The lumbar lordosis (LL) was defined as the angle between the upper endplate of L1 and the superior endplate of S1 (Positive when in lordosis). The pelvic incidence (PI) corresponded to the angle between the perpendicular to the sacral plate at its midpoint and the line connecting the same point to the center of the bi-coxo-femoral axis. The pelvic tilt (PT) was defined as the angle between the line connecting the middle of the superior endplate of S1 to the center of the bi-coxo-femoral axis and the vertical line. The sacral slope (SS) corresponded to the angle between the sacral plate and the horizontal plane. The osteotomized vertebral angle (OVA) was defined as the difference between the angle formed by the upper and lower endplates of the osteotomized vertebrae on full-length, standing sagittal radiographs at pre-operation and final follow-up (Positive when OVA towards the ventral).

According to previous literatures reported [13], the calculation methods of theoretical spino-pelvis parameters were as follows: tPT $=$ PI $\times$ 0.37 $-$ 7°, tLL $=$ PI $\times$ 0.54 $+$ 32°, tSS $=$ PI $-$ tPT, tTK $\le$ tLL $-$ 20°, SVA $\le$ 5 cm.

### Preoperative prediction method of osteotomy angle

Through literature review, the widely recognized CAM-HA method, FBI method, SFA method, Surgimap method, and HP-HA method were evaluated in this study.

#### FBI method

**Fig. 2:**
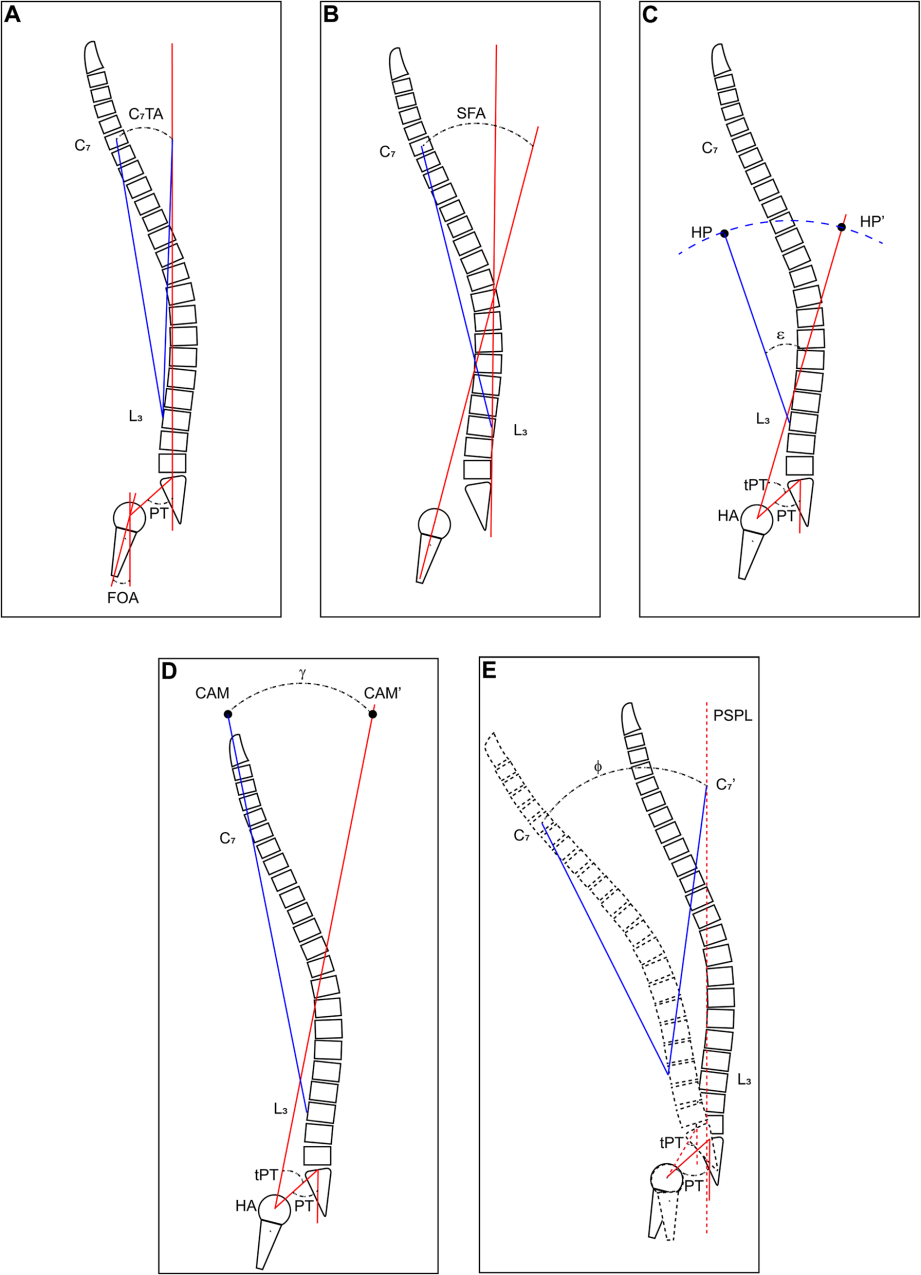
Schematic diagrams demonstrating for the calculation methods of various methods. **a** FBI method, the osteotomy angle $\alpha =$ C_7_TA $+$ FOA $+$ CTPA; **b** SFA method, the osteotomy angle $\beta =$ SFA $+$ ITK $+10$; **c** HP-HA method; **d** CAM-HA method; **e** Surgimap method

The FBI technique was equal to the sum of angle of C7 translation (C7TA), angle of femur obliquity (FOA), and angle of pelvis tilt compensation (PTCA). C7TA referred to the angle formed by transposing the midpoint of C7 vertebrae horizontally on the plumb line ascending from the mid part of the S1 plate, with the osteotomy vertebra as the axis of rotation. FOA was measured as the angle between the femoral axis and the plumb line. If PT between 15 $^\circ$ and 25 $^\circ$, PTCA $=$ 5 $^\circ$; if PT superior 25 $^\circ$, PTCA $=$ 10 $^\circ$ (Fig. 2a).

#### SFA method

Needed correction angle $=$ SFA $+$ 10 $^\circ +$ ITK. SFA was the angle formed by the femoral axis and the line drawn from the center of C7 to the point where the plumbline from of the posterior end of the S1 plate intersecting the level of the planned osteotomy. The angle of 10 $^\circ$ represented the hip extension reserve. ITK was a postoperative increase in thoracic kyphosis and calculated as the difference between active flexion thoracic kyphosis (ATK) and standing thoracic kyphosis (TK) (Fig. 2b).

#### HP-HA method

The postoperative individual PT was defined firstly by the mathematical equation: PT $=$ 0.37 $\times$ PI $-$ 7. Second, a line passing through HA and the midpoint of S1 was drawn, and the postoperative plumb line was drawn according to the calculated PT. Next, a circle was drawn by taking the apex of the selected osteotomy site as the center, and the distance between this point to the HP as radius. Lastly, the circle was intersecting the plumb line and the included angle was measured and used as the osteotomy angle (Fig. 2c).

#### CAM-HA method

The theoretical PT (tPT $=$ 0.37 $\times$ PI $-$ 7) was used to set the neutral position of the pelvis. Then, the apex of the selected osteotomy site served as the center and the distance between this point to the CAM was regarded ad radius. Lastly, CAM was rotated to the plumb line of HA and the included angle was used as the osteotomy angle (Fig. 2d).

Surgimap method: the full-length, standing lateral radiograph was rotated to make PT equal to or less than 20 $^\circ$ using Surgimap software. Then, the apex of the selected osteotomy site was served as the center and the distance between this point to the center of C7 vertebrae was regarded as radius. Lastly, the center of C7 vertebrae was rotated to the plumb line ascending from the posterior corner of S1 and the included angle was used as the osteotomy angle (Fig. 2e).

### Statistical analysis

Statistical analysis was conducted using the SPSS version 19.0 (SPSS Inc., Chicago, IL, USA). All results were expressed as mean $\pm$ standard deviation ($\overline{x }\pm s$). The paired *t* test was used to evaluated differences among preoperative, theoretical, and postoperative parameters, and between the predictive osteotomy angle and the OVA. Statistical significance was reached at *P* $<$ 0.05.

## Results

### Demographic data

All 22 patients (18 males and 4 females) were enrolled in this study. The average age was 39.30 $\pm$ 12.31 years old (18 $\sim$ 60 years old). The mean follow-up time was 30.00 $\pm$ 3.56 months (24 $\sim$ 36 months). All patients received posterior single-level PSO osteotomy treatment, and the selected osteotomy site was at T12 in three patients, L1 in six patients, L2 in nine patients, and L3 in four patients. The preoperative sagittal parameter measurements of all patients were listed in Table 1.

**Table 1:** Demographic and characteristics of AS patients

	AS patients
Gender (F/M)	4/18
Age (ys)	39.30 $\pm$ 12.31
Follow-up (m)	30.00 $\pm$ 3.56
The osteotomy site	
T12	3
L1	6
L2	9
L3	4
PI ($^\circ$)	48.57 $\pm$ 9.24
PT ($^\circ$)	37.93 $\pm$ 5.37
SS ($^\circ$)	10.60 $\pm 9$.02
LL ($^\circ$)	$-$ 9.95 $\pm$ 14.14
TK ($^\circ$)	37.27 $\pm$ 21.49
SVA ($\mathrm{cm}$)	19.21 $\pm$ 5.04
OVA ($^\circ$)	46.57 $\pm$ 2.32

### Theoretical spino-pelvis parameters

Table 2 summarized the theoretical spino-pelvis sagittal parameters in 22 AS patients according to the reported calculation methods. The preoperative and theoretical parameters were found to be significantly different in terms of SVA (19.21 $\pm$ 5.04 $^\circ$ vs. 5.00 $\pm$ 0.00 $^\circ$), PT (37.93 $\pm$ 5.37 $^\circ$ vs. 10.92 $\pm$ 3.42 $^\circ$), SS (10.60 $\pm$ 9.02 $^\circ$ vs. 37.65 $\pm$ 5.82 $^\circ$), and LL ($-$ 9.95 $\pm$ 14.14 $^\circ$ vs. 53.8 $\pm$ 4.16 $^\circ$). No significant difference was found between preoperative and theoretical TK (37.27 $\pm$ 21.49 $^\circ$ vs. 32.4 $0\pm$ 5.10 $^\circ$, *P* $>$ 0.05).

**Table 2:** Comparison among preoperative, theoretical, and postoperative parameters

	Preoperative values	Theoretical values	Postoperative values	*P*	*P	**P
PI ($^\circ$)	48.57 $\pm$ 9.24	-	47.69 $\pm$ 8.85	-	0.110	-
PT ($^\circ$)	37.93 $\pm$ 5.37	10.92 $\pm$ 3.42	22.78 $\pm$ 7.60	0.000	0.000	0.001
SS ($^\circ$)	10.60 $\pm 9$.02	37.65 $\pm$ 5.82	24.91 $\pm$ 9.93	0.000	0.000	0.000
LL ($^\circ$)	$-$ 9.95 $\pm$ 14.14	53.8 $\pm 4.16$	40.14 $\pm$ 10.99	0.000	0.000	0.001
TK ($^\circ$)	37.27 $\pm$ 21.49	32.4 $0\pm$ 5.10	41.65 $\pm$ 13.04	0.518	0.549	0.056
SVA ($\mathrm{cm}$)	19.21 $\pm$ 5.04	$\le$ 5	5.50 $\pm$ 3.26	0.000	0.000	0.000

*P*, preoperative data vs. theoretical data using paired *t* test

^*^P, preoperative data vs. postoperative data using paired *t* test

^**^P, theoretical data vs. postoperative data using paired *t* test

### Comparison among preoperative, theoretical, and postoperative parameters

Table 2 summarized the sagittal parameters at final follow-up in 22 AS patients. Significant statistical differences were observed in terms of PT, SS, LL, and SVA compared to pre-operation (*P* $<$ 0.05). No significant difference was found between the postoperative and preoperative TK (41.65 $\pm$ 13.04 $^\circ$ vs. 37.27 $\pm$ 21.49 $^\circ$, *P* $>$ 0.05). The postoperative and theoretical values were found to be significantly different in term of PT, SS, and LL (*P* $<$ 0.05).

### Comparison between the predicted angle and the osteotomized vertebral angle

Surgimap software was used to measure the osteotomy angle according to various methods (Table 3). Comparing with the osteotomized vertebral angle (46.57 $\pm$ 2.32 $^\circ$) at final follow-up, the angle predicted by Surgimap method (53.80 $\pm$ 9.79 $^\circ$), HP-HA method (60.07 $\pm$ 13.58 $^\circ$), and CAM-HP method (56.61 $\pm$ 8.58 $^\circ$) had a significantly higher value (*P* $<$ 0.05). However, no significant difference was found between the osteotomized vertebral angle and those of FBI (48.08 $\pm$ 12.49 $^\circ$) and SFA (51.24 $\pm$ 12.14 $^\circ$) method (*P* $>$ 0.05).

**Table 3:** Comparison of the OVA with the osteotomy angles predicted by different methods

Method	Osteotomy angle ($^\circ$)	*P* value
SFA method	51.24 $\pm$ 12.14	0.226
HP-HA method	60.07 $\pm$ 13.58	0.008
CAM-HA method	56.61 $\pm$ 8.58	0.003
Surgimap method	53.80 $\pm$ 9.79	0.042
FBI method	48.08 $\pm$ 12.49	0.704

*P*, the osteotomy angle by various methods vs. the OVA at final follow-up using paired *t* test

## Discussion

As the condition progresses, AS patients may develop a series of postural changes, such as, reduced lumbar lordosis, pelvis back-tilt, hip hyperextension, knee flexion, and cervical flexion deformity, affecting the global trunk balance. Spinal kyphosis deformity is a prominent feature of these patients, usually leading to shift the center of gravity of the trunk anteriorly. It was reported that about 30% of AS patients without standard conservative treatment may develop thoracolumbar or lumbar kyphosis [2]. In order to restore the balance of the trunk, a series of compensatory mechanisms will be initiated [14]. When the anterior sagittal imbalance of the spine excesses the compensatory capacity of the body, AS patients will lose the ability of lying down flat, walking, and horizontal visual axis, and have impaired function of the respiratory and digestion systems resulted from compressed visceral organs in severe cases [15]. To improve the quality of life in such patients, surgical intervention is an indispensable treatment protocol.

Osteotomy is widely used to correct spinal deformity and improve health-related quality of life in AS patients with thoracolumbar or lumbar kyphosis [16]. Among them, pedicle subtraction osteotomy is a common procedure to improve postoperative sagittal parameters. Due to the specialty and complexity of ankylosing spondylitis, however, the relationship between the optimal postoperative sagittal parameters and HRQOL remains obscure. Schwab et al. [17] reported that SVA, PT and PI $-$ LL were three most important parameters for surgeons to make an operative proposal in adult spinal deformity, furthermore, proposed that postoperative SVA $<$ 5 cm, PT $<$ 25 $^\circ$, and PI $-$ LL $=\pm$ 9 $^\circ$ were favorable indicators for good clinical prognosis. Kim et al. [18] also reported that SVA was an important prognostic index for AS patients with rigid kyphosis deformity, undergoing posterior osteotomy procedure. The authors indicated that patients with postoperative SVA less than 70 mm were able to obtain better ODI scores (17.4 $\pm$ 8.2). Recently, Huang et al. [19] analyzed the clinical data of AS patients with thoracolumbar kyphosis deformity treated by single-level PSO procedure, and proposed that postoperative PT $<$ 24 $^\circ$, SSA $>$ 108 $^\circ$ and TPA $>$ 152 $^\circ$ indicated better clinical outcomes (ODI $<$ 20). In addition, Lee et al. [20] found that cervical sagittal alignment in AS patients was different from that of normal population, and obviously related to HRQOL. In present study, all patients’ ODI scores were less than 20 and the value of SVA decreased from 19.21 $\pm$ 5.04 cm preoperatively to 5.50 $\pm$ 3.26 cm postoperatively. In addition, compared to preoperative parameters, postoperative PT, SS, and LL were significantly improved. These data indicated that all patients enrolled in this study achieved good clinical outcomes and deformity correction in treatment with posterior single-level PSO.

Currently, the osteotomy correction of rigid sagittal deformity remains a tricky procedure. The reason is, partly, that it is difficult to accurately quantify the osteotomy angle required to restore optimal sagittal alignment before surgery. In 2006, Ondra [6] and Yang [7] proposed a trigonometric method for calculating osteotomy size of PSO to correct fixed sagittal deformity. In this method, the vertex of the selected osteotomy segment was used as the rotation axis, and the included angle formed by the rotation and translation of the center of C7 vertebrae to the plumb line ascending from the posterior corner of S1 was the osteotomy angle. Because of insufficient correction due to ignoring the compensatory role of pelvis and lower limbs, this method has been rarely used in clinical practice. Then, van Royen et al. [21] designed a computational program for preoperative planning in AS patients and defined the normal sacral endplate angle at 40 $^\circ$. Although it integrated the role of pelvis compensation, but the compensatory effect of lower limbs did not be considered. Furthermore, defining the postoperative SEA at 40 $^\circ$ was ambiguous and unreasonable, especially in patients with small PI.

In the past decade, with the deepening understanding the global balance, scholars have proposed different calculation method of the osteotomy angle. But the effectiveness and rationality of various methods need to be further evaluated. From a biomechanical standpoint, the ideal method for maintaining sagittal balance is to shift the center of gravity (CG) of the trunk over the hip axis when the pelvic and lower extremity joints are in the neutral position. In the sagittal plane, the center of both acoustic meati overhang almost coincides with the center of mass of head, and the whole spine is well-balanced if the CAM overhang is less than or equal to 2 cm. Based on abovementioned findings, Aurouer et al. [8] proposed a CAM-HA method for preoperative planning for correction of sagittal deformity of the spine. The authors normalized the value of PT according to the formula of tPT $=$ 0.37 $\times$ PI $-$ 7, then simulated osteotomies to make CAM overhang less than 2 cm. In 2013, Song et al. [12] found that the hilus pulmonis was located over HA in normal subjects, then proposed the HP-HA method for calculating the osteotomy angle. In this method, the postoperative individual PT was identified similar to that of CAM-HA method, then the HP was served as the CG of the upper trunk to calculate the angle of osteotomy by taking the HP through the plumb line of HA.

Both of two methods, though, take the compensatory effects of pelvis and lower extremity into account, but there still exist some drawbacks. As we know, ankylosing spondylitis generally starts from the sacroiliac joint and gradually erodes the spine cephalad to cranio-cervical junction region. In different stages of disease progression, the thoracic and cervical region can retain a certain amount of mobility. Ankylosing spondylitis with thoracolumbar or lumbar kyphosis leads to lean forward, often accompanied by a compensatory decrease in thoracic kyphosis and a compensatory increase in cervical lordosis or C2-7 SVA, which may be spontaneously corrected after osteotomy surgery. In addition, the position of CAM is subject to variation with that of head, and the high quality of full spine radiographs covering the landmark of CAM are sometimes difficult to obtain, all of which contribute to the inaccuracy of osteotomy angle calculation by CAM-HA method. Although the center of gravity of the trunk is replaced with HP, which eliminates the influence of body position, in HP-HA method, but the anatomical variation of the hilus pulmonis and the unclear image due to overlapped soft tissue structures decrease its location accuracy in X-ray. Second, the position of HP is usually located at the level of T4 vertebrae, so it is not suitable for patients with relatively high osteotomy site. In this study, the osteotomy angles predicted by CAM-HA method (56.61 $\pm$ 8.58 $^\circ$) and HP-HA method (60.07 $\pm$ 13.58 $^\circ$) were significantly greater than the actual angle (46.57 $\pm$ 2.32 $^\circ$) at final follow up, which were usually not achieved only by a single-level PSO procedure.

In 2011, Le Huce et al. [9] proposed a full balance integrated (FBI) technique for osteotomy planification of thoracolumbar imbalance. The following factors were included in this method: C7TA, FOA, and PTCA, which took both effects of pelvis and lower extremity into account. In terms of PTCA, the authors suggested that if the PT was less than 25 $^\circ$ or more than 25 $^\circ$, 5 or 10 $^\circ$ of PT compensation should be added, respectively, by experience. However, Lamartina et al. [10] thought that FBI method just roughly estimated the amount of pelvic tilt excess and lack of consideration of thoracic hypo-lordosis, therewith, proposed a new method to avoid potential drawbacks of FBI method, while keeping its simplicity. This method is based on the measurement of a single angle, the spino-femoral angle (SFA). Beyond that, the hip extension reserve (10 $^\circ$) and increase in thoracic kyphosis after surgery are taken into consideration. Recently, Akbar et al. [11] offered a process in corrective osteotomy surgery with respect to the calculation of the osteotomy angle needed via using Surgimap software. The desired postoperative PT can be reached by rotating the image, next bring C7 in line with the posterosuperior corner of S1, then the resection angle needed can be measured by Surgimap Spine software. Our results showed that no significant difference was found between the osteotomy angle of SFA method (51.24 $\pm$ 12.14 $^\circ$) and FBI method (48.08 $\pm$ 12.49 $^\circ$) and the actual angle at final follow up, but the angle of Surgimap method (53.80 $\pm$ 9.79 $^\circ$) was slightly larger than the actual osteotomy angle.

In fact, the PSO can result in approximately 30 degrees of correction with maximum bony resection when performed at the apex of a sharp deformity [22]. Therefore, for AS patients without need for performing two-level PSO procedure, the SPO at adjacent levels may be served as a supplement to obtain the desired outcome of osteotomy. Second, both pre-bending of titanium rod and enhanced fixation by internal fixation system have an influence on postoperative sagittal alignment. Therefore, combining experimental results with our experiences, SFA, FBI, and Surigmap method are all suitable for calculating the osteotomy angle preoperatively in AS patients with thoracolumbar or lumbar kyphosis, however, considering the simplicity and rationality of all three methods, SFA may be superior to the others.

This study was associated with several limitations. First, AS patients enrolled in this study were characterized by thoracolumbar or lumbar kyphosis. The results of this study are not applicable to AS patients with cervical deformity or flexion contracture deformity of the hip. Second, this study was retrospective in nature. Finally, the sample size was small, and multicenter, large sample, and the long-term clinical observations are needed to confirm this conclusion.

## Conclusion

In AS patients with thoracolumbar or lumbar kyphosis, who plan to perform a posterior single-level PSO procedure, the SFA, FBI, and Surgimap method can all be used to predict the osteotomy angle required for obtaining optimal postoperative sagittal balance. However, considering the simplicity of application and the rationality of design, the SFA method is more suitable for such population.

## Data Availability

All data generated or analysed during this study included in this published article.

## Abbreviations

AS: Ankylosing spondylitis
HRQOL: Health-related quality life
SPO: Smith-Peterson osteotomy
PSO: Pedicle subtraction osteotomy
CAM-HA: Center of both acoustic meati-hip axis
FBI: Full balance integrated technique
SFA: Spino-femoral angle
HP-HA: Hilus pulmonis-hip axis
SVA: Sagittal vertical axis
TK: Thoracic kyphosis
LL: Lumbar lordosis
PI: Pelvic incidence
PT: Pelvic tilt
SS: Sacral scope
OVA: Osteotomized vertebral angle
C7TA: Angle of C7 translation
FOA: Angle of femur obliquity
PTCA: Angle of pelvis tilt compensation
ITK: Increase in thoracic kyphosis
ATK: Active flexion thoracic kyphosis
